# Zebrafish exposure to environmentally relevant concentration of depleted uranium impairs progeny development at the molecular and histological levels

**DOI:** 10.1371/journal.pone.0177932

**Published:** 2017-05-22

**Authors:** Olivier Armant, Kewin Gombeau, Sophia Murat El Houdigui, Magali Floriani, Virginie Camilleri, Isabelle Cavalie, Christelle Adam-Guillermin

**Affiliations:** Institut de Radioprotection et de Sûreté Nucléaire (IRSN), PRP-ENV/SERIS/LECO, Cadarache, Saint-Paul-lez-Durance, France; Laboratoire Arago, FRANCE

## Abstract

Uranium is an actinide naturally found in the environment. Anthropogenic activities lead to the release of increasing amounts of uranium and depleted uranium (DU) in the environment, posing potential risks to aquatic organisms due to radiological and chemical toxicity of this radionucleide. Although environmental contaminations with high levels of uranium have already been observed, chronic exposures of non-human species to levels close to the environmental quality standards remain scarcely characterized. The present study focused on the identification of the molecular pathways impacted by a chronic exposure of zebrafish to 20 μg/L of DU during 10 days. The transcriptomic effects were evaluated by the use of the mRNAseq analysis in three organs of adult zebrafish, the brain the testis and the ovaries, and two developmental stages of the adult fish progeny, two-cells embryo and four-days larvae. The results highlight generic effects on the cell adhesion process, but also specific transcriptomic responses depending on the organ or the developmental stage investigated. The analysis of the transgenerational effects of DU-exposure on the four-day zebrafish larvae demonstrate an induction of genes involved in oxidative response (*cat*, *mpx*, *sod1* and *sod2*), a decrease of expression of the two hatching enzymes (*he1a* and *he1b*), the deregulation of the expression of gene coding for the ATPase complex and the induction of cellular stress. Electron microscopy analysis of skeletal muscles on the four-days larvae highlights significant histological impacts on the ultrastructure of both the mitochondria and the myofibres. In addition, the comparison with the transcriptomic data obtained for the acetylcholine esterase mutant reveals the induction of protein-chaperons in the skeletal muscles of the progeny of fish chronically exposed to DU, pointing towards long lasting effects of this chemical in the muscles. The results presented in this study support the hypothesis that a chronic parental exposure to an environmentally relevant concentration of DU could impair the progeny development with significant effects observed both at the molecular level and on the histological ultrastructure of organs. This study provides a comprehensive transcriptomic dataset useful for ecotoxicological studies on other fish species at the molecular level. It also provides a key DU responsive gene, *egr1*, which may be a candidate biomarker for monitoring aquatic pollution by heavy metals.

## Introduction

Chemical risk assessment is conducted on animal models based on regulatory test guidelines with the key objective to assess a relationship between the concentration of exposure and the associated biological effects observed to determine the concentration of substance above which adverse effects can be expected. Usually, high concentrations are used to detect adverse endpoints such as lethality and reproductive effects. Evidence has been accumulated showing that high-dose effects can be different from low-dose effects. For example non-monotonic dose-responses and threshold effects can be observed [1,2]. In addition, the chronic exposures of individuals to low concentrations of pollutants have also the potential to alter the health of the offspring with deleterious effects that can be transmitted over generations through the inheritance of genetic or epigenetic alterations in the progeny of the exposed individuals [3,4]. As such, how the effects observed at the individual level can spread over generations and impact the population scale remains uncertain for a vast majority of pollutants.

Uranium is an actinide that can be found in the environment at concentrations in surface and ground waters ranging from 0.01 to more than 2000 μg/L [5,6]. Environmental quality standard for uranium range from 0.3μg/L to 30μg/L depending on the physico-chemical properties of the water (pH, hardness and dissolved organic matter content) [7]. Natural uranium is composed of three isotopes: 99.27% is ^238^U (half-life of 4 billion years), 0.72% ^235^U (half-life 700 million years) and less than 0.001% ^234^U (half-life 427000 years). DU shows a decreased ^235^U percentage down to 0.2%. It is a main by-product of the nuclear fuel cycle, it is used as counterweights in the aircraft industry, in biological shielding for radiation protection and is used as armour penetrator in military conventional ammunitions. High levels of natural [8] and depleted uranium [9] have already been observed, either in the vicinity of former mining sites or after accidental spillage of industrial sites. With the growing world nuclear energy demand, DU concentration in the geosphere is expected to increase in the next decades [10], raising concerns on the long-term effects of this chemical on the environment. DU is a heavy metal chemically toxic but weakly radioactive. Studies realized so far on animals and human for acute exposures point towards nephrotoxic [11] and carcinogenic effects of DU [12,13]. At the molecular levels, *in vitro* assays on cultured cells showed that acute exposure to DU can alter gene expression and induce oxidative stress as well as double-strand DNA breaks [14,15].

Few studies investigated the biological impairments induced over generations by a chronic parental exposure to environmentally relevant concentrations of DU. The chronic effects of DU started to be investigated *in vivo* on the zebrafish, *Danio rerio*, a species that is gaining interest in the modeling of human diseases, including cancer, and recommended by the OECD guideline for the testing of chemicals. Exposure of fertilized eggs to 20 and 250 μg DU/L during 20 days was associated with a significant decrease of the hatching rate and survival during development [16]. The increase of oxidative stress upon uranium exposure was shown to induce the expression of detoxifying genes like *sod1*, *sod2* and *cat* in the liver [17]. Mitochondrial and DNA repair enzymes were also perturbed in zebrafish skeletal muscles and brain [17,18], similarly to what was observed in the kidney of mice chronically exposed to uranium [19]. DU bioaccumulates in the gonads of females chronically exposed and is transmitted to the eggs [20,21]. This transfer from the exposed adult to the progeny was proposed to account for the genotoxic effects of uranium. Finally, we demonstrated recently that DU-exposure altered the transcriptome and the global DNA methylation levels in adult tissues and in the progeny (F1 generation) of the DU-exposed fish [22].

In the present study, we decided to go deeper in the analysis of the transcriptomic changes induced by chronic exposure to DU on zebrafish, using data generated previously [22] and by producing novel data. We performed a detailed analysis of the DU-induced transcriptomic changes by mRNAseq on adult brain and gonads (testis and ovaries), as well as on the progeny of the DU-exposed fish at two-cells stage and 96 hours post fertilization (hpf). A comparison with the transcriptome of the acetylcholine esterase mutant, where a progressive degeneration of the muscles fibre damages is observed [23], reveals similar induction of protein chaperons in response to stress (such as *hsp70l* and *hsp70*) in skeletal muscles. In addition to this global analysis of the transcriptome, we attempted to link the DU-induced transcriptional changes with the histopathological injuries observed by transmission electronic microscopy (TEM) in the muscles of 96 hpf larvae, thereby providing potential molecular mechanisms for the observed physiological effects.

## Material and methods

### Fish maintenance and depleted uranium exposure

All zebrafish work was carried out with the approval from the Animal User and Ethical Committee at the IRSN (committee 81). Care was taken to minimize the numbers of animals used in these experiments in accordance with the ARRIVE guidelines. Adult wild type zebrafish (*Danio rerio*, AB genetic background) 6–9 months of age were purchased from Amagen (Gif-sur-Yvette, France). Thirty males (average wet weight of 0.47 ±0.05 g and 3.57 ±0.13 cm) and females (average wet weight of 0.69 ±0.16 g and 3.78 ±0.16 cm) were maintained in 30L glass tanks containing synthetic soft water (CaCl_2_[2H_2_O] 42.49 mg/L, MgCl_2_[6H_2_O] 19.30 mg/L, MgSO_4_[7H_2_O] 24.65 mg/L, Na_2_CO_3_ 0.78 mg/L, KCl 11.33 mg/L, and NaNO_3_ 26.35 mg/L) and oxygenated by air bubbling. The composition of the synthetic water was chosen to allow the maximal bioavailability of uranium, as previously described [24]. The temperature was set to 28°C ± 1°C, pH to 6.5 ± 0.1 through the experiment and under a day light cycle of 14h/10h (day/night). Fish were fed twice a day with standard fish flakes (Tetramin, Melle, Germany) and once a day with 24hpf *Artemia salina nauplii* (JBL, Herblay, France). The males and females were maintained separated and crossed once per week in synthetic soft water during the 3 weeks of acclimatization and then exposed to a concentration of 20μg/L DU (UO_2_(NO_3_)_2_ − 6H_2_O, Sigma, Lezennes, France). DU concentration was checked several times per day [20] to ensure that the contamination was as closed as possible from the nominal concentration. The direct quantification of DU in the tanks showed that the actual DU concentration was 16.5 ± 2.9 μg/L for ten days. On the 6^th^ days all males and all females from DU exposed and control groups were mated in clean water (one couple per tank) and the eggs allowed to develop until 96 hpf in clean water. Following the breeding, the adult fish were replaced in DU contaminated water for a total time of 10 days. Fish behaviour and feeding were observed daily during the whole time of the experiment to detect any sign of distress or suffering. No death, behavioural differences or sign of suffering were observed in the DU-exposed fish group compared to controls. All fish were euthanized at the end of the experiment by immersion in ice cold water. Measurement of body mass and length didn’t reveal any difference between the exposed and control group (data not shown). Dissection of brain, ovaries and testis was conducted under the binocular (Leica, France). The main entry of uranium in the body is through the gills [25]. The DU-bioaccumulation in brain, gonads, two-cells stage embryos and 96 hpf larvae was analysed on mineralized samples using a 7500Cx spectrometer (ICP-MS, Agilent) with a detection limit of 0.11 ng/L. A concentration of 1.55 ±1.11 μg U/g of dry tissue was detected in the brains of exposed fish, 0.92 ±0.4 μg U/g of dry tissue in the testis, 2.31 ±1.52 μg U/g of dry tissue in the ovaries, 4.51 ±0.94 μg U/g of dry tissue in the two-cells stage embryos and 0.56 ±0.09 μg U/g of dry tissue in the 96 hpf larvae. More details on the methods can be found in the companion paper [22].

### mRNAseq expression analysis

Total RNA samples were extracted using the Absolutely RNA Miniprep kit (Agilent) according to manufacturer’s recommendations from whole gonads of adult males and females, and from pools of 50 embryos at two-cells stages and three larvae at 96 hpf. Sequencing libraries were prepared from 1μg of total RNA with TruSeq mRNA version 2 kits from Illumina following manufacturer’s instructions. No signs of degradation were detected on RNA nano Chips (Bionalyser 2011, Agilent). Libraries were multiplexed at 8pM and run on a Hiseq1500 to produced 2x51bp or 2x70bp paired end reads. Reads were mapped against the Zv10 *Danio rerio* genome assembly with RNA-STAR[26], using the exon-exon junctions from Ensembl (release 85). A total of 3 billions of high quality paired end reads (Q>30) were generated (S1 Table). The assessment of biological replicates quality by hierarchical clustering led to the selection of 22 samples (S1 Fig). Data normalization and differential expression analysis were performed with DESeq2[27]. Genes with a log2 fold change > ±1 and adjusted *p*-value (FDR) < 0.01 were considered as differentially expressed. Hierarchical clustering was performed in R with the *hclust* package using Pearson correlation and the complete linkage method on a set of 6140 genes with rlog normalized expression (from DESeq2) consistently > = 11 in the biological replicates for at least one condition within the following condition: brain, testis, ovaries, two-cells stage embryos, 96 hpf larvae. Five-ways Venn-diagram was constructed with BEG online tools (http://bioinformatics.psb.ugent.be/cgi-bin/liste/Venn/calculate_venn.htpl) and the *p*-value for significance of the overlap computed by a hypergeometric test.

### Gene Ontology and diseases analysis

Gene Ontology (GO) enrichment analysis were performed on zebrafish Ensembl identifiers with the R package *TopGO* [28]. Unique human orthologues were collected from *biomart* when the % of identity between the zebrafish and human genes was > 30%. Zebrafish and human gene-set provided similar enrichment of GO terms with *TopGO* or Gene Set Enricher available from CTD website (http://ctdbase.org/tools/enricher.go) (S5 Table and data not shown). To infer the diseases enrichment, human orthologues were submitted to the CTD Enriched diseases tool (http://ctdbase.org/tools/enricher.go). GO pathways and MESH terms (disease names) with *p*-values < 0.01 and < 0.001 respectively (exact Fisher’s test) were considered as significantly enriched. Upstream regulators were identified using human orthologues as a query to ChEA2 (http://amp.pharm.mssm.edu/ChEA2).

### Comparison to other mRNAseq datasets

Fastq files from mutant zebrafish for the gene *acetyl choline esterase* (ache-/-) (72 hpf larvae) were downloaded from GSE74202 [29]. Alignments to the ZV10 genome assembly and differential expression analysis were performed as described above. Genes with adjusted *p*-value < 0.01 were used for significance of differential gene expression.

### Microscopic analyses

The 96 hpf larvae were fixed with 2.5% (w/v) glutaraldehyde in 0.1 M, pH 7.4 sodium cacodylate buffer for two days at 4°C. The samples were washed three times 5 min in the same buffer. Samples were treated to 1% (w/v) osmium tetroxyde in cacodylate buffer for 1h, then dehydrated through a graded ethanol series, and finally embedded in monomeric resin (Epon 812). All chemicals used for histological preparation were purchased from Electron Microscopy Sciences (Hatfield, USA). Sections for optical and electron microscope of 500 and 80 nm respectively were obtained with an ultramicrotome UCT (Leica Microsystems GmbH, Wetzlar, Germany). The semi-thin sections were stained with aqueous blue toluidine and examined under a light microscope (Leica, DM750) equipped with a Leica camera ICC50 and the LAS EZ Software. For TEM analysis, ultrathin sections were mounted on copper grids and examined in a Tecnai G^2^ Biotwin Electron Microscope (FEI Company, Eindhoven, the Netherlands) using an accelerating voltage of 100 kV and equiped with a CCD camera Megaview III (Olympus Soft imaging Solutions GmbH, Münster, Germany). For each replicate, at least 20 micrographs of local detailed structures were taken, analysed and compared for each condition.

### Quantitative RT-PCR

Reverse transcription was performed from 50–100 ng of total RNA obtained from two different pool of F1 96 hpf larvae and controls (biological duplicates), with SuperScript III (Invitrogen) and oligo(dT) primers in final volume of 20 μl following instructions from the manufacturer. Quantitative PCR (qPCR) was performed on *egr1*. Probes specific for *egr1* were designed using the Primer3 website [30]. Target primer sequences were forward 5’-TCACCTTGCTGGAGATACGC-3’, reverse 5’-CACTCACCAGGCTGAACAGA-3’. Similarly to previous studies *β-actin* was found to be the most consistent and least variable control for this analysis [31]. The primer sequence for *β-actin* was the same as used in a previous study [32] forward 5’-CTAAAAACTGGAACGGTGAAGG-3’ and reverse 5’-AGGCAAATAAGTTTCGGAACAA-3’. qPCR was performed on a Mx3000P Real-Time PCR Detection System (Stratagene, Agilent) using the Brillant III Ultra-Fast SYBR Green QPCR Master Mix (Agilent), forward and reverse primers diluted at 0.25 μM (final concentration), and ROX-dye diluted to 30 nM in a final volume of 20 μl. The cycling parameters included 1 cycle of pre-incubation at 95°C for 5 minutes, then 45 cycles of amplification at 95°C for 15 s, 60°C for 20s and 72°C for 40 s and a final step for melting curve analysis at 95°C for 1 minute, 55°C for 30 s and 95°C for 30 s. Experimental samples were run in duplicates (technical replicates) and gene expression was normalized to *β-actin* using the ΔΔCt method.

## Results

### Molecular effects on adult tissues after chronic DU-exposure

**Fig 1:**
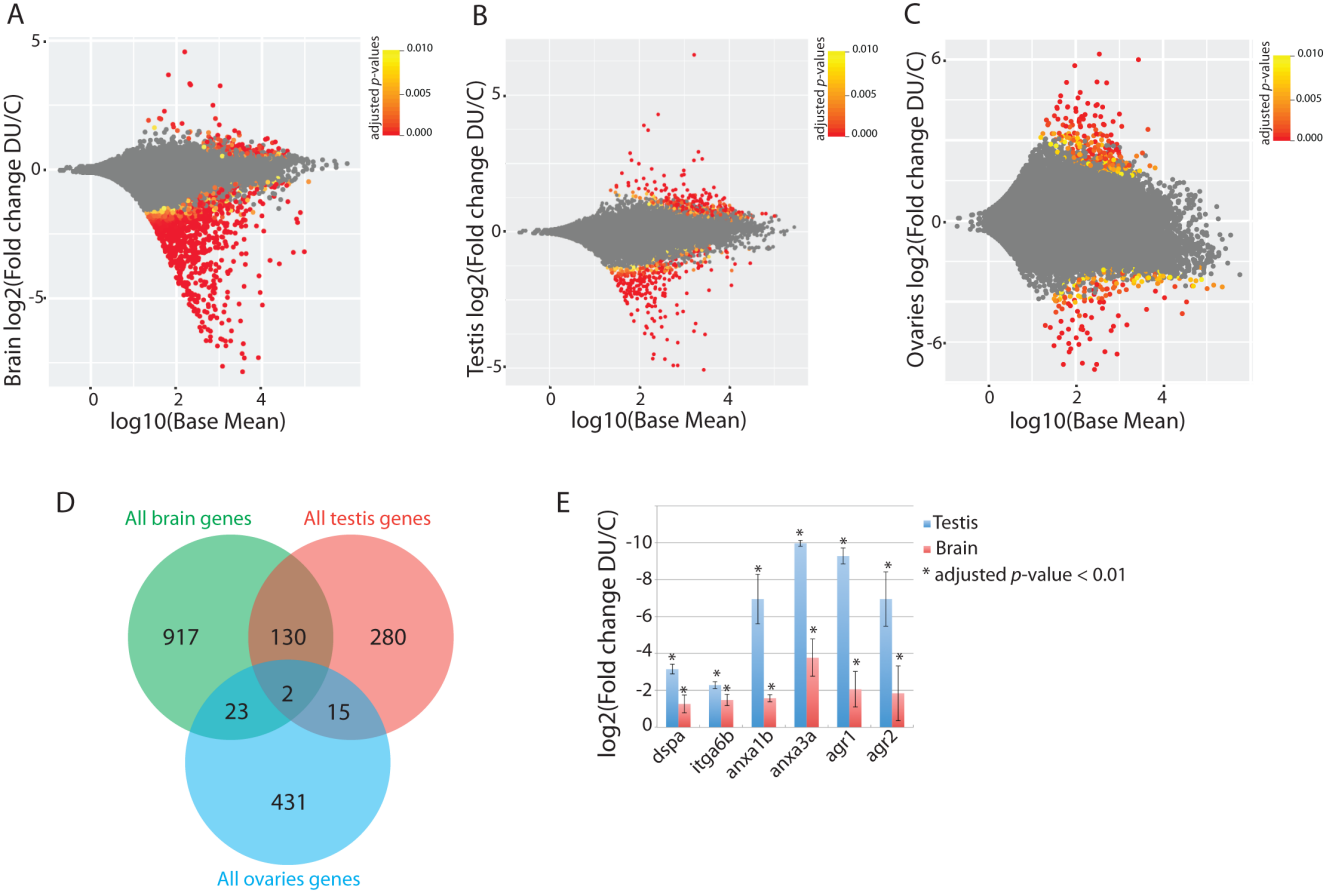
Differential gene expression analysis in the adult tissues following a 10 days chronic exposure to DU. (A-C) MA-plot for the differential analysis in the adult brain (A), testis (B) and ovaries (C). Expression for each gene is shown on the x-axis as the log10 of the mean normalized expression, and the log2 of the fold change on y-axis. Significant adjusted *p*-values (FDR) < = 0.01 are highlighted in yellow to red, and non-significant changes in grey. (D) Venn diagram of genes mis-regulated in the brain, testis and ovaries. All genes significantly regulated (up- or down- regulated) were used to compare the three tissues. (E) Fold change of genes involved in regeneration and cell adhesion in DU exposed brain and testis compared to controls (all adjusted *p*-values (FDR) < 0.01; error-bars represent the standard deviation to the mean).

**Fig 2:**
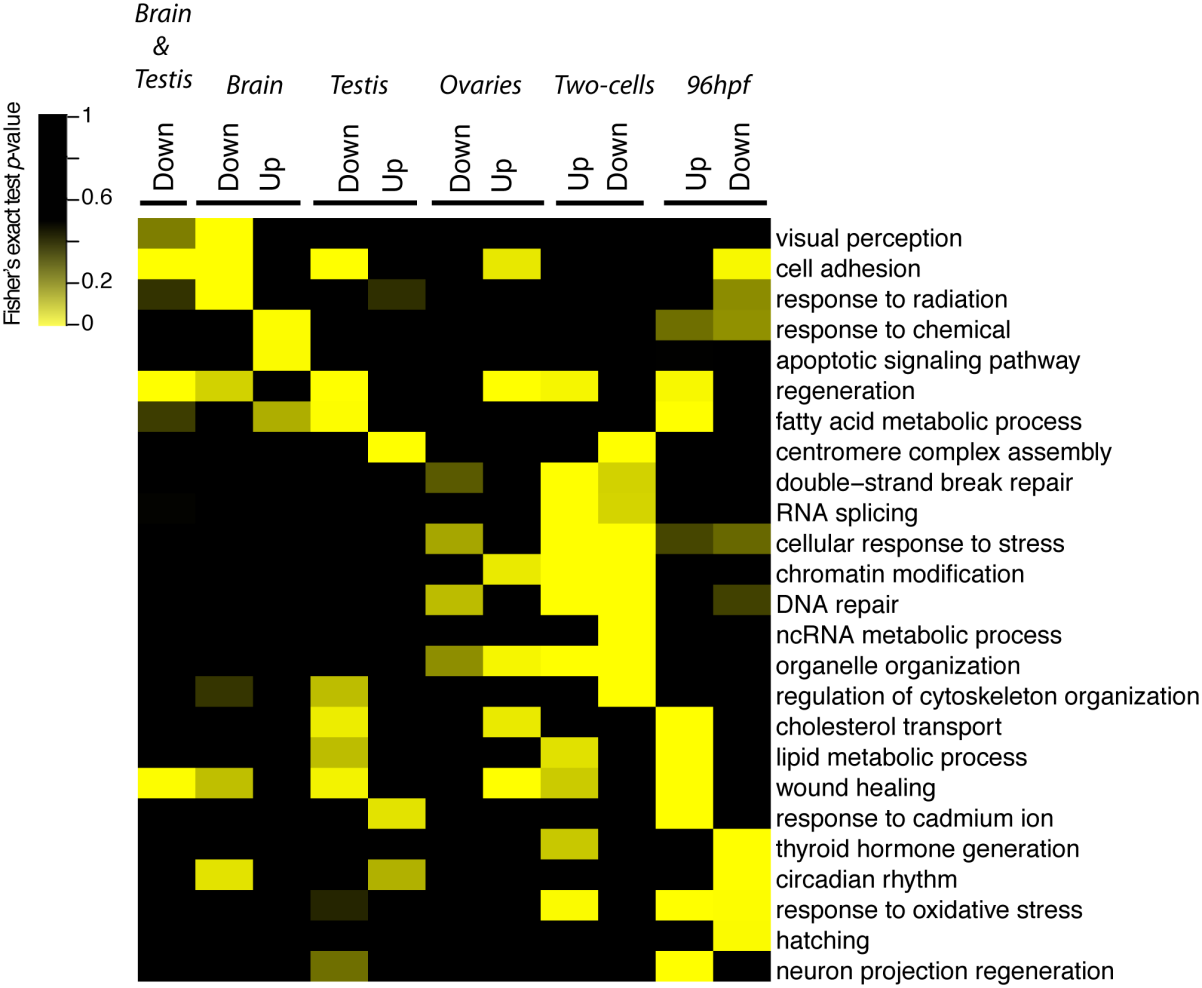
Heatmap of GO terms enriched in DU-exposed adult’s tissues and their progeny. The *p*-values from Fisher’s exact-test are indicated, as well as the associated GO terms. The datasets of up- and down-regulated genes used for the pathways analysis are indicated on the top of the heatmap (relative to fold change as DU exposed/control). The set of genes down-regulated in both adult brain and testis is also indicated. Yellow: Fisher’s exact test *p*-values < 0.01; black: non-significant enrichment.

We performed a global analysis of the transcriptomic changes induced by DU by mRNAseq on adult brain and adult gonads (testis and ovaries), as well as on the F1 progeny of exposed adult zebrafish. The mRNAseq analyses were performed in three biological replicates that generated a total of 3 billions of high quality paired end reads (Q>30) (S1 Table, S1 Fig). To get insights into the effects of DU on adult tissue homeostasis, we performed a differential gene expression analysis. In the brain, we detected 1072 genes significantly mis-regulated following DU-exposure (log2 fold change > ±1 and FDR < 0.01). Most of the genes (*n* = 1026) were down-regulated as compared to controls, while only 46 genes were up-regulated (Fig 1A). The analysis of the signalling pathways based on Gene Ontology (GO) enrichment indicated that the down-regulated genes were mainly involved in visual perception, cell adhesion, and response to radiation, while the up-regulated genes were involved in inflammation, response to chemical and apoptosis (Fig 2 and S2 Table). In the testis, the chronic exposure to DU triggered the up-regulation of 132 genes and the down-regulation of 295 genes (Fig 1B). The genetic pathways involved in cadmium response and centromere assembly were significantly enriched in the set of up-regulated genes. Signalling pathways involved in regeneration, lipid metabolism and cell adhesion, were significantly enriched in the group of down-regulated genes (Fig 2, S2 Table). In the ovaries, we detected the up-regulation of 302 genes and the down-regulation of 169 genes (Fig 1C). We noted that the control samples had a higher variability (S1 Fig) which limits this analysis to the most significant differential gene expression between the exposed and the control groups. The GO-term analysis showed that up-regulated genes were involved in cell adhesion, regeneration, oocyte production and in several regulatory pathways involved in organ development, including the nervous system development (S2 Table). The down-regulated genes were mainly involved in androgen biosynthetic process and mitophagy.

The comparison of all regulated genes (up or down-regulated) in all three adult tissues (brain, testis and ovaries) highlighted only two genes regulated in common: *desmoplakin a* (*dspa*), involved in cell adhesion and the gene *si*:*dkey-193p11*.*2* of unknown function (Fig 1D). A significant overlap was observed between the brain and the testis (132 genes, *p* < 10^−29^, Fig 1D). Only one gene (*zgc*:*153426*) was up-regulated both in the exposed brain and the testis, while 125 genes were down-regulated in both tissues (corresponding to 10.5% of the total number of genes detected in both tissues, *n* = 1196) (S2 Fig). Pathways enrichment analysis showed that these down-regulated genes were involved in cell adhesion (*dspa*, *integrins*, *anexins*) and regeneration (*agr1*, *agr2*) (Figs 1D, 1E and 2, S2 and S3 Tables). These data show that DU has tissue specific effects on brain, ovaries and testis, but also more general effects on adult tissues including cell adhesion and tissue regeneration.

### Transgenerational effects of DU-exposure

**Fig 3:**
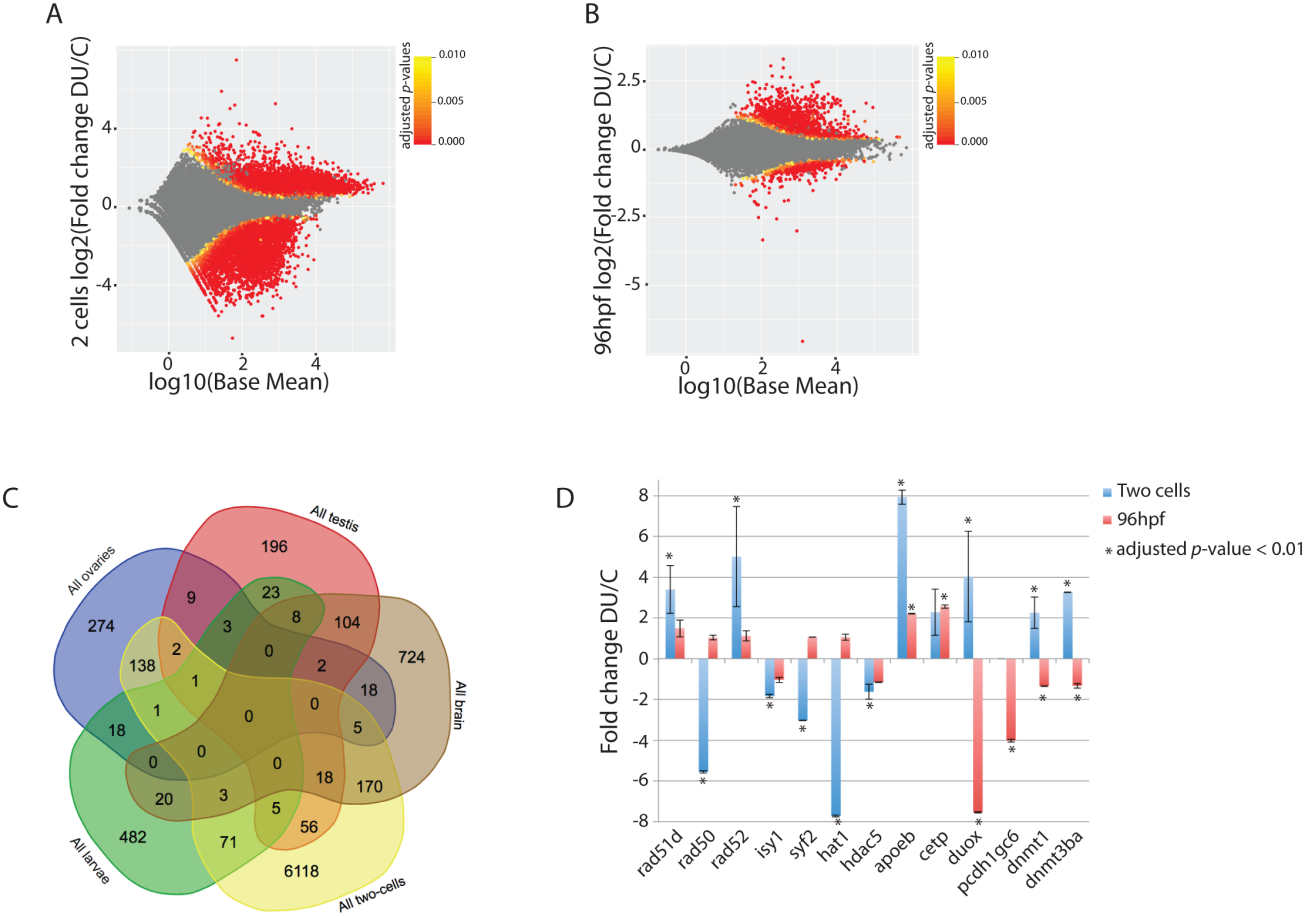
Differential expression analysis in two-cells stage embryos and 96 hpf larvae obtained from DU-exposed adult fish. (A) MA-plot at the two-cells stage. (B) MA-plot at 96 hpf. (C) Five-ways diagram of all genes regulated in adult brain, testis, ovaries and in the progeny of DU-exposed fish at two-cells stage and 96 hpf. (D) Examples of expression changes in the progeny of DU-exposed fish of genes involved in DNA repair (*rad51d*, *rad50*, *rad2*), splicing (*isy1*, *syf2*), chromatin remodelling (*hat1* and *hdac5*), lipid transport (*apoeb*, *cetp*), oxidative stress (*duox*) and cell adhesion (*pcdh1gc6*) (all adjusted *p*-values (FDR) < 0.01; error-bars represent the standard deviation to the mean).

To get insights on the potential molecular impact of DU parental exposure, we first analysed the transcriptome of the two-cells stage embryos, before the maternal-zygotic transition occurs near 6hpf (shield-stage)[33]. The parental exposure to DU induced change in the expression of 6588 genes, comprising 2482 up-regulated genes and 4106 down-regulated genes (Fig 3A). The GO terms enrichment analysis pointed towards global effects on many different pathways at the two-cells stage (S4 Table). Effects on DNA repair (*rad51d*, *rad50*, *rad52*), RNA splicing (*isy1*, *syf2*), chromatin remodelling (*hdac5*, *hat1*), maturation of ncRNA and cell division were also detected (Figs 2 and 3D and S4 Table). After four days of development (ie. 96 hpf larvae) the number of mis-regulated genes dropped to 573 and 62 genes respectively up- and down-regulated (Fig 3B). The analysis of GO terms based on the set of up-regulated genes revealed a significant enrichment of pathways involved in cholesterol transport (*apoeb*) and response to oxidative stress (*cat*, *sod1*, *sod2*, *mpx*), while pathways involved in cell adhesion (*pcdh1gc6*) and hatching (*he1a* and *he1b*) where enriched in the down-regulated genes (Figs 2 and 3D). Only 81 genes (1.1%, *n* = 7142) were regulated in common between the two-cells stage and 96 hpf larvae (Fig 3C), the genes being either up- or down-regulated in this comparison.

In order to find potential commonalities in the effects of DU on the different organs and developmental stages, we compared to transcriptomics changes in the brain, the gonads, the two-cells stage embryos and in 96 hpf larva. The only significant overlap between all conditions remained the brain and testis (Fig 3C). Little overlap was found for the other conditions. For instance, only 82 genes (1.2%, *n* = 7015) were mis-regulated in common (up or down) between the adult testis and the two-cells stage, 40 genes (3.8%, *n* = 1062) between adult testis and 96 hpf larvae, 31 genes (1.8%, *n* = 1707) between brain and 96 hpf larvae, 196 genes (2.6%, *n* = 7660) between the brain and the two-cells stage, 147 genes (2.1%, *n* = 7059) between the ovaries and the two-cells stage, 17 genes (1.9%, *n* = 898) between the ovaries and the testis, and finally only 23 genes (2.1%, *n* = 1106) between the ovaries and the 96 hpf larvae. No genes were mis-regulated in common in all five conditions. These results show that the molecular signature of DU is mostly different in the different adult tissues and in the progeny of exposed fish. It further suggests that the early global effects observed in the two-cells embryos are partially compensated later in development.

### Predicted diseases and transcription factors networks perturbed by DU

The results of the transcriptomic analyses were processed to identify the potential diseases occurring after chronic exposure to DU. Human gene orthologues to the deregulated zebrafish genes were selected and provided similar GO term enrichments to the zebrafish analysis (see Material and methods and S5 Table). The most significant disease predicted in all five conditions was cancer (S6 Table). A significant enrichment (*p* < 10^−20^) of genes implicated in genetic/congenital and metabolic diseases was also observed for the embryonic stages (two-cells stage and 96 hpf larvae) but was less significant (or absent) in adult tissues (S6 Table). We found that 60% of the diseases (68 out of 113 Medical Subject Headings, MESH terms) significantly enriched (*p* < 0.001) at 96 hpf were also enriched at the two-cells stage (S3A Fig). But despite this overlap the number of genes implicated in biological disorders and co-regulated at two-cells and 96 hpf remained low (2%) (S3B Fig and S7 Table). These results suggest that although the different transcriptomic patterns observed by mRNAseq at the two-cells stage and the 96 hpf stage are different, they nonetheless converge towards the same disease effects.

We then carried out an analysis of the transcriptional regulators potentially controlling the differentially expressed genes. In this analysis the known gene regulation networks were used to identify the transcriptional regulators that better explain the differential expression observed in the mRNAseq datasets. This identification of upstream-regulators (transcription factors and chromatin remodelers) was performed using the human orthologous genes to benefit from the rich functional annotations of the human genome. Four transcription factors, EGR1, HNF4A, MITF and FOXA2, appeared to form a hub of upstream regulators in all five gene-sets (*p* < 0.001) and could explain the apparent overlap in disease outcomes (ie. cancer). The molecular signature of the two TGF-B downstream effectors SMAD2 and SMAD3 was highly enriched both in the brain and testis, while GATA1, GATA2 and ESR1 were significantly enriched only in 96 hpf larvae (S8 Table). Of note, the expression of two of these master regulators was altered in the mRNAseq data, while this is actually not a prerequisite for the analysis of upstream-regulators (S9 Table). Quantitative RT-PCR analysis confirmed that *egr1* expression was upregulated in the 96 hpf larvae from DU-exposed fish (S4 Fig). These results show that DU-exposure has broad effects on cell function in adult zebrafish as well as on their offspring, potentially leading to metabolic disorders and cancer. These effects might be caused mainly by the perturbation of the genetic networks controlled by egr1, hnf4a, mitf and foxa2.

### Transgenerational effects of chronic exposure to DU on mitochondrial functions

**Fig 4:**
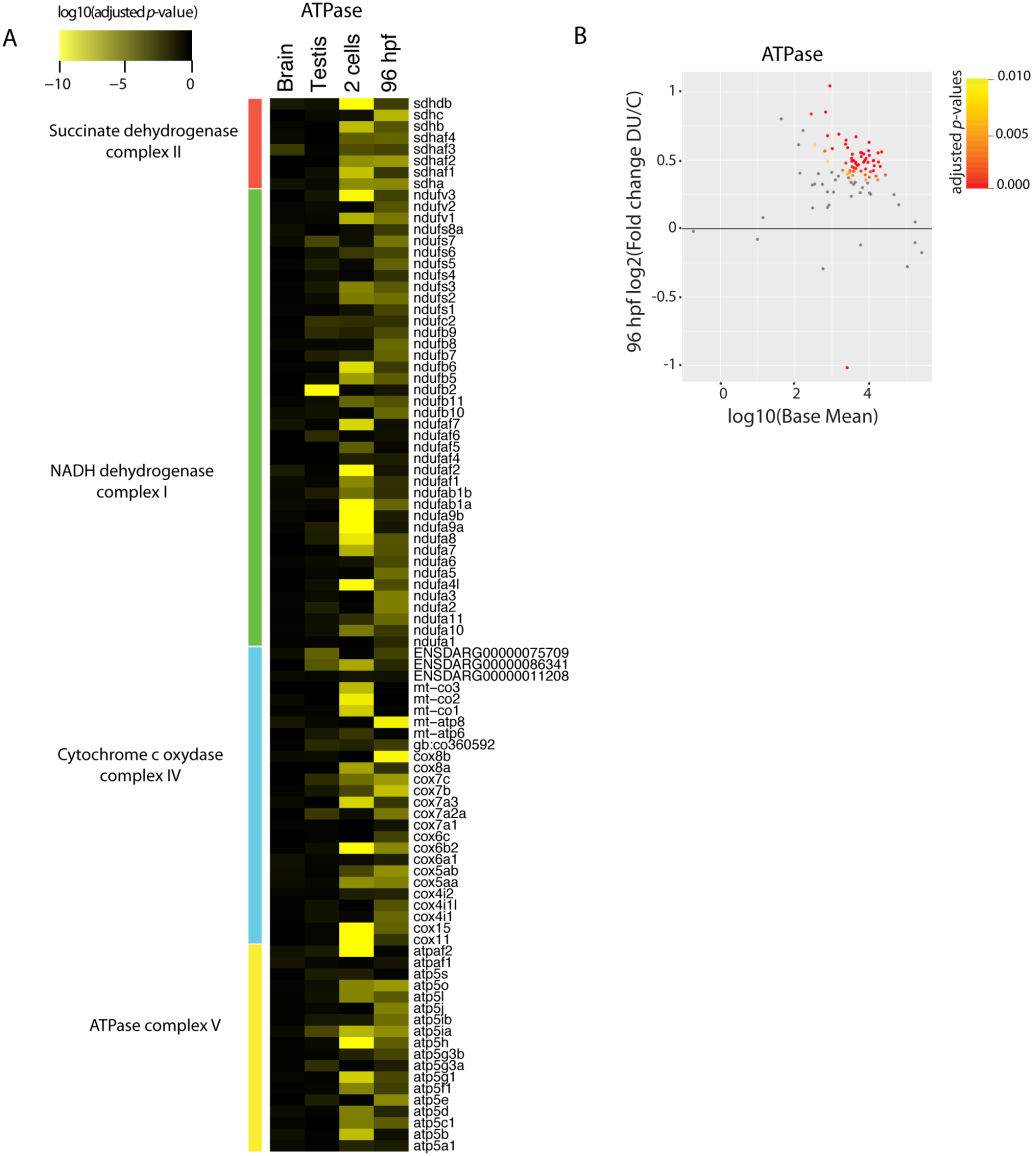
Expression analysis of electron transport chain complex genes in testis and brain of DU exposed fish and in their progeny at two-cells stage and 96 hpf. (A) Heatmap of adjusted *p*-values of genes involved in mitochondrial oxydo-reduction process (*n* = 101). The colour code displays the log10(adjusted *p*-value) from the differential expression analysis. (B) MA-plot focusing on the differential expression of the electron transport chain complex genes in the 96 hpf larvae (fold change as DU/C). Most of the genes are up-regulated in DU exposed larva.

**Fig 5:**
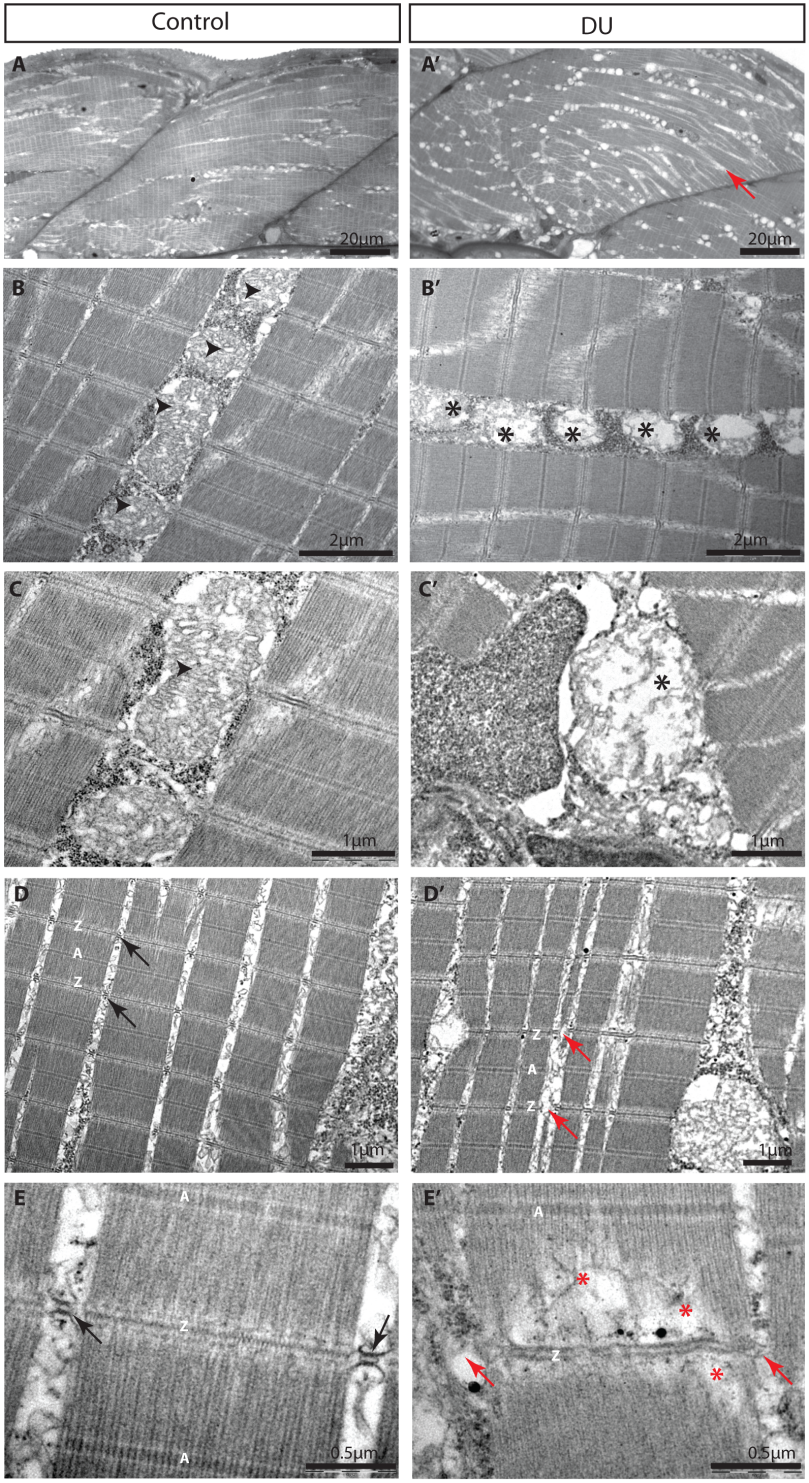
Histological analysis of 96 hpf larvae from DU-exposed adult fish compared to controls. (A-A’) Toluidine-blue staining of the larva at the level of the trunk. Vacuole-like structures (appearing as white dots) are abundant in DU-treated larvae compared to controls. (B-C) Mitochondrial morphology in skeletal muscles observed by transmission electronic microscopy of control larvae. The inner mitochondrial membranes are dense and well visible (black arrow head). (B’-C’) Alteration of mitochondrial morphology and absence or decreases density of inner mitochondrial membranes (*) in larvae from DU-exposed fish. (D-E) High-resolution transmission electronic microscopy of skeletal muscles in controls and (D’-E’) in the 96 hpf larvae obtained from DU-exposed fish. Disruption of myofibres (red asterisks) and swelling of Z-bands are visible in DU larvae. Z- and A-bands are indicated. Triads constituted of t-tubes and the two flanking cisterna of the sarcoplasmic reticulum are visible in the controls (black arrows) but are absent or deformed in larvae from DU-treated fish (red arrows).

The transcriptomic analyses performed on the two-cells stage and in 96 hpf larvae revealed an alteration of the pathways involved in fatty acid metabolism and response to oxidative stress. Many genes in these two pathways were up-regulated (Fig 2 and S4A and S4B Fig). As the mitochondria play a central role in redox homeostasis and the metabolism of lipids, we hypothesized that the induction of these two pathways could be a consequence of mitochondrial dysfunctions, as it was already shown for adult zebrafish exposed chronically to DU [18]. To assess this hypothesis we selected 101 genes encoding the subunits of the electron transport chain complex as a read out of mitochondrial function and checked their expression at the two embryonic stages (two-cells and 96 hpf), thereby focusing on the transgenerational effects of DU-exposure. The results highlighted an up-regulation of the majority of the genes producing the subunits of the respiratory chain complex I, II, IV and V at both developmental stages (Fig 4A and 4B), suggesting that mitochondria function was perturbed in the progeny of DU-exposed fish. We then performed histological analysis on the skeletal muscles of the 96 hpf larvae from DU-exposed adult fish to check potential mitochondrial abnormalities. Muscle was chosen since this organ is rich in mitochondria producing the energy necessary for muscular contraction. Vacuole-like structures were apparent compared to controls (Fig 5A–5A’) suggesting a deep alteration of the skeletal muscles. A more detailed analysis by transmission electronic microscopy (TEM) revealed abnormal mitochondria exhibiting fewer or completely disrupted inner membranes (Fig 5B’–5C’, black asterisks).

### Chronic exposure to DU result in a disruption of muscle fibers in the progeny of exposed fish

The TEM observations performed on the skeletal muscles of 96 hpf larvae from DU-exposed fish revealed a high frequency of sarcomer disruptions that resulted in the fragmentation of the muscles fibres (Fig 5A’, red arrow). The observation of the myofibrils at higher resolution pointed out to a swelling of the Z-lines and a detachment of the myofibres (black arrows and red asterisk in Fig 5D’–5E’). In addition, the t-tubules and the two flanking cisterna of the sarcoplasmic reticulum that form the triads were also malformed or even missing (red arrow head in Fig 5D’–5E’). Since muscle contraction is dependent on the depolarization of the sarcolemma-derived t-tubules and the transmission of Ca^2+^ flux to the sarcoplasmic reticulum at the triads, the neuromuscular transmission is presumably altered in the muscles of 96 hpf larva obtained from DU-treated adult fish. In agreement with this hypothesis, several genes involved in neuronal projection regeneration were stimulated in 96 hpf larvae of DU-treated fish (Fig 2 and S4B Fig) including *dpysl2b*, *epha4b*, *cntn2*, *tnc*, *clcf1*, and the proneural genes *neurog1* and *ascl1a*.

**Fig 6:**
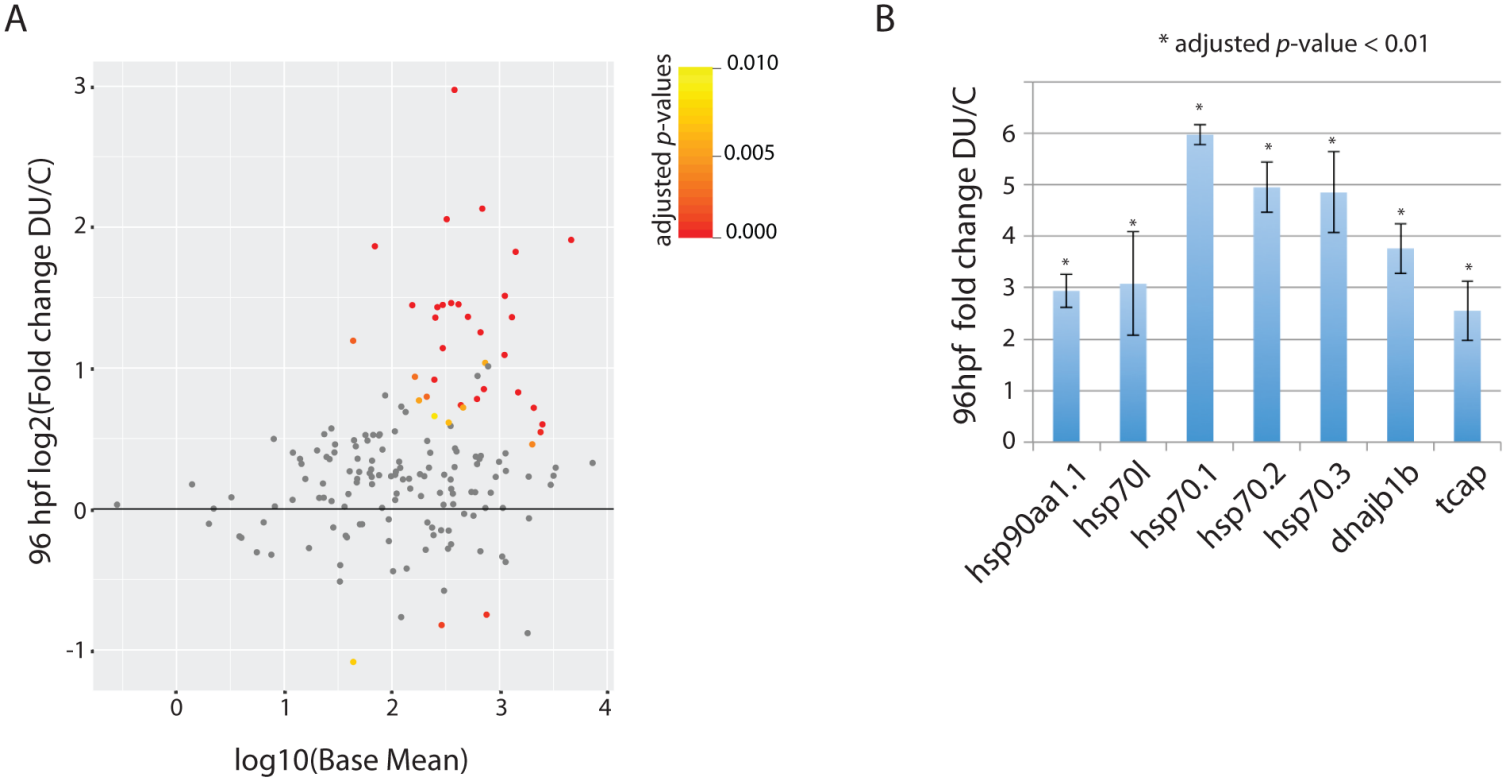
Induction of protein chaperones expression following myofibre damage in the 96 hpf larvae from DU-exposed adult zebrafish. (A) MA-plot focusing on the differential expression of the 169 genes involved in cellular stress (from the ache mutant, see Material and methods) and their regulation in 96 hpf larvae obtained from DU exposed. Significant adjusted *p*-values < 0.01 are indicated in red and yellow. (B) Log2 fold change of specific protein-chaperon in the skeletal muscles (all adjusted *p*-values (FDR) < 0.01; error-bars represent the standard deviation to the mean).

It is already known that protein-chaperons like *hsp70* and *hsp90aa1*.*1* are induced after myofibres damages [34,35]. To check if similar mechanisms are in play we compared the data from 96 hpf larva of DU-exposed fish with the transcriptomics changes observed in the zebrafish *acetylcholine esterase* (*ache*) mutant (ache-/-) [23]. A progressive degeneration of the myofibres is observed in ache-/- during development due to ache receptor over-activation, a situation related to myofibres damages where the induction of *hsp70l* and *hsp70* is also observed. We found that among the 169 genes up-regulated in ache-/-, 38 genes (22%) are also mis-regulated in the 96 hpf larvae from DU-treated fish with an adjusted *p*-value < 0.01 (Fig 6A), including the up-regulation of the protein chaperons *hsp70l*, *hsp70*.*1*, *hsp70*.*2*, *hsp70*.*3*, *dnajb1b* (*hsp40*) and the gene coding for the sarcomere assembly protein *tcap* (*titin-cap*) (Fig 6B).

## Discussion

In this study we investigated the consequences of chronic exposure to DU on adult zebrafish and their progeny. The adults were exposed for 10 days to a DU concentration of 16.5 ± 2.9 μg/L (see Material and methods), a concentration that is in the range of the environmental quality standards for uranium in water (< 30μg/L) [36,37]. The macroscopic observation and feeding behaviour of the exposed fish (made accordingly to the guidelines, see S10 Table) was normal compared to controls. We tested the transcriptomic effects of DU on adult tissues including brain and gonads. The progeny of DU-exposed fish was also analysed at the two-cells stage and on 96 hpf larvae. The methodology of sampling, the results of DU quantification during the experiment as well as the bioaccumulation of DU in the different organs were already published in the companion paper [22]. This analysis focuses on the mRNAseq data obtained from the DU-exposed adults’ testis, ovaries, brain and the F1 progeny at the two-cells stage and 96 hpf.

We were first interested in the identification of the generic and tissue specific molecular signature following the DU-exposure. The analysis of the adult ovaries shows that 471 genes are differentially expressed. However, only the most significant differential expressed genes are detected in this analysis due to a probable higher inter-individual differences in the number and stage of germ cells present in the females. In the brain, we find that the genetic pathway involved in visual perception is affected. The tectum is the part of the diencephalon that processes the visual input from the retina in vertebrates. The tectum and the retina are functionally coupled by topographical projections of retinal axons to the tectum and many transcription factors are simultaneously expressed in both tissues during development [38]. Interestingly an alteration of the outer plexiform layer was observed in the retina of DU exposed fish [22]. Since the tectum process the visual input from the retina, it might be possible that the damages induced by DU in the retina modify the transcriptomic pattern in this brain area. Alternatively, DU could directly impact the function of the optic tectum, independently from its effects on the retina.

The data obtained on testis highlighted an effect of DU-exposure on centromere assembly, the process that direct chromosome segregation during cytokinesis. Two genes involved in this pathway were up-regulated in the DU-exposed testis: centromere protein O and a CENP-A containing nucleosome assembly gene (*si*:*dkey-148h10*.*5*). These two genes were down-regulated significantly in the two-cells stage embryos. Interference with this biological process leads to the uneven segregation of chromosome during cell division and results to daughter cells with different number of chromosomes (aneuploidy). DU is known to induce sister chromatin exchanges, chromosomal aberrations and DNA double-strand breaks, effects that are collectively called genomic instability [39]. In addition, it has been shown that the DU-induced oxidative stress can induce aneuploidy in *in vitro* genotoxic assays [40,41]. As the gametes are produced continuously in the testis, the number of germ cells undergoing cell divisions is very high in this organ. The effects observed in the testis could thus be a consequence of the increased occurrence of centromeric defects leading to chromosomal aberrations in this highly proliferating tissue. Both effects in the adult tissues, perturbation of visual perception in brain and centromeric defects in the testis could alter with time the capabilities of the DU-exposed fish to survive and to reproduce in a contaminated environment.

We found a specific transgenerational effect of DU on the hatching process on the four-days old larvae. The two hatching enzymes *he1a* and *he1b* were still down-regulated in the 96 hpf larvae obtained from DU-exposed fish, after the onset of hatching (near 48 hpf). Interestingly, a precedent study showed that the hatching rate was delayed by 42% when zebrafish embryos were grown in 250μg/L DU [16]. More analysis will be necessary to follow the dynamic of expression of he1a and he1b in presence of increasing DU-concentration and the potential impact on hatching. Hatching delays could also be caused by developmental delays. A detailed analysis of the morphological abnormalities in the larvae obtained from DU-exposed fish was not done here, but the observed decreased expression of the hatching enzymes in this study could explain the effects on hatching rate.

The direct comparison of the transcriptomics changes induced by DU between the five mRNAseq datasets showed no overlap, which suggests that the molecular response of DU in the different stages and tissues are mostly different. The comparison between the brain and the testis was the most significant with 132 genes regulated in common. Like the brain, the testis is a lipid-rich organ and both organs are immunologically privileged, which could explain some similarities in the response to DU [42,43]. The pathways analysis on the 125 genes down-regulated in common in both organs showed significant enrichment of the biological process of cell adhesion and regeneration. Cell adhesion was also down-regulated in 96 hpf larva obtained from DU exposed fish, showing that this process may also be affected in embryos that were not directly exposed to DU. It is possible that DU itself can be transmitted from the chronically exposed females to the eggs during oogenesis thereby explaining the long lasting effects of DU observed in the 96 hpf larva. In favour of this hypothesis, precedent analysis on chronically exposed adult zebrafish showed that DU bioaccumulates in the ovaries and eggs of females [18,22]. However, we did not observe an alteration of cell adhesion at the two-cells stage embryos which suggests that this pathway is either not significantly affected by DU at the transcript levels or that this pathway is not transcriptionally active at this stage. In agreement with the latter, E-cadherin, the major adhesion protein active during cleavage stages in zebrafish, is maternally expressed [44] and thus not transcribed actively before the maternal-zygotic transition occurs. It is thus possible that DU affects cell adhesion in a generic manner in adult organs and on larvae but that this effect is not detected by mRNAseq in two-cells stage embryos, because this pathway is not yet actively transcribed.

Interestingly, cell adhesion is also inhibited by cadmium, another divalent heavy metal like uranium [45] (uranium is under the UO_2_^2+^ form in water) and we also observed a significant activation of the pathway involved in cadmium response in the 96 hpf larvae and in the adult testis. Cadmium, uranium and other heavy metals have nephrotoxic effects [46]. In addition, uranium and cadmium were recently proposed to induce oxidative stress and to produce similar histopathological effects in the hepatopancreas of the crayfish [47]. These results highlight possible similarities in the toxicological mechanism of these two heavy metals. Cell adhesion and apoptosis are important pathways involved in the mechanisms of toxicity of cadmium [45,48]. In addition, the detachment of cells from the cellular matrix lead to cell apoptosis and cell adhesion itself is deregulated during carcinogenesis. Our data support the hypothesis that DU and cadmium may induce carcinogenesis through similar mechanism of toxicity, including the perturbation of cell adhesion.

*In vitro* studies have demonstrated that uranium can induce DNA double-strand breaks and inhibit the activity of DNA-PK kinase activity involved in the non-homologous end-joining (NHEJ) reparation system, thereby decreasing the abilities of cells to repair DNA [49], and hence potentially lead to mutations and cancer. Recent data also demonstrate the modification of epigenetics marks after chronic exposure to DU [22] that could contribute to the transgenerational and the carcinogenic effects of DU. DU can induce tumorigenesis in osteoblast cell lines [50] but epidemiological [51] and *in vivo* studies are scarce [12]. Our data suggest that, upon chronic exposure, low levels of DU can affect cell adhesion and exerts its genotoxic effects, in the long term, in a way that is, at least in part, reminiscent of the mechanism of action of cadmium, which might involve the induction of DNA mutations and carcinogenesis. However, the hypothesis suggesting that the decrease of cell adhesion and the induction of carcinogenesis are central to explain the toxicity of low concentrations of DU will need further investigation.

The analysis of upstream regulators potentially regulating the differentially expressed genes points out four transcription factors, egr1, hnf4a, mitf and foxa2, that appear to form a hub of upstream regulators explaining a substantial part of the DU-induced transcriptomics changes. The global changes observed at the two-cells stage could account for the enrichment of egr1 and the cancer pathway at this stage. Nevertheless, the power of this analysis allows to highlight similarities in the effects of DU on all five conditions that are missed when we focus only on the differentially expressed genes. The gene egr1 (early growth-response 1) encodes a C2H2 type zinc-finger transcription factor involved in cell adhesion, proliferation and apoptosis [52]. A decrease of the expression or the deletion of the EGR1 gene in human is linked to the occurrence of non-small cell lung carcinoma and breast carcinoma, while its activation has growth-inhibiting and suppressing roles in tumorigenesis [53]. In addition EGR1 directly regulates the expression of the cell adhesion protein KRT18 [54], providing a possible molecular link between the observed alteration of cell adhesion process in our mRNAseq data and the prediction that cancer is the most statistical relevant disease outcome in the five conditions tested. Importantly, Egr1 is also involved in the cellular response to oxidative stress [55]. Indeed, data from *in vitro* studies demonstrated that elevated redox levels in the cells induce the nuclear translocation of the DNA repair enzyme APE1 which stabilizes EGR1 DNA binding activity through protein-protein interactions [56]. In line with an activation of the *egr1* oxidative stress pathway in zebrafish, we observed an enrichment of the transcriptomic response to oxidative stress in the larvae from DU-exposed fish. The central role of EGR1 in several key biological effects of DU toxicity makes this transcription factors a highly relevant biomarker. The similarity in the DU and cadmium biological effects suggests that *egr1* could report the toxic activity of other heavy metals.

Electronic microscopy analyses and mRNAseq data allowed us to correlate the induction of oxidative stress to a probable dysfunction of mitochondrial activities. Indeed, the expression of the ATPase complex was induced after exposure to DU probably reflecting a potential compensatory mechanism in response to the loss or the disruption of inner mitochondrial membranes observed by TEM. A decrease of mitochondrial respiration and change in the expression of the cytochrome oxydase have already been reported in the muscles and brain of adult zebrafish chronically exposed to 30 μg/L DU during 10 and 28 days [18], and are coherent with our results on the 96 hpf larvae obtained from DU-exposed fish. These data collectively suggest that the mechanism of DU toxicity might include the perturbation of the mitochondrial activity. Mitochondria dysfunction will result in an increased oxidative stress, inducing DNA-damages and in the long run, could lead to the accumulation of mutations that will favour, in the longer term, cell apoptosis and carcinogenesis. Our study highlights the role of *egr1* in the translation of these molecular effects at the cellular level, but more analyses will be needed to prove that *egr1* is a key biomarker of DU toxicity.

Another aspect of DU toxicity predicted from the transcriptomic data relates to the perturbation of the genetic networks regulated by *hnf4a* and *foxa2*. These two transcription factors synergise to regulate the transcription of the genetic pathways involved in the intermediary metabolism of glucose, fatty acid and cholesterol. Mutations or dysregulations of the expression of either gene lead to metabolic disorders [57]. We found an up-regulation of fatty acid metabolism in the exposed brain and in 96 hpf larvae from DU-exposed fish, while this pathway was down-regulated in the adult testis. Why this pathway is differentially regulated in the testis is not clear, but our results are in agreement with previous studies in rodent that showed a modification of the cholesterol metabolism in the brain of adult rat exposed to DU [58]. In addition to the effects on adult, our data also highlighted a perturbation of the cholesterol metabolism in the offspring of DU-exposed fish. The analysis of potential DU-induced diseases highlights a significant enrichment of metabolic disorders (*p* < 10^−20^) in the embryos and larvae obtained from DU exposed fish. Based on these findings it is probable that parental DU-exposure impacts the health of the offspring, at least in part, by inducing disorders linked to the metabolism of lipids. If these effects are independent from oxidative stress and mitochondrial dysfunction induced by DU remains to be determined.

Our study also revealed that chronic exposure to a low concentration of DU can alter skeletal muscles integrity in the progeny of exposed fish. TEM analysis showed a high level of vacuolization in the muscles of four-days old larvae, as shown previously [18], a detachment of myofibres from the Z-lines, as well as a disruption of the sarcoplasmic reticulum at the triads that may impact neurotransmission at the neuromuscular junction. The transcriptomics analysis performed in the 96 hpf obtained from DU exposed fish revealed that many protein chaperons *hsp70*, *hsp70l* and *dnajb1b* were up-regulated. Several zebrafish mutants exhibit similar up-regulation of chaperons due to the degeneration or detachment of myofibres as for instance in the *ache* zebrafish mutant [23]. We observed that 22% of the 169 genes up-regulated in *ache* mutant were also altered in DU-exposed larvae. The mechanisms of action of DU on myofibres are not known and are perhaps not related to what is observed in the *ache* mutant. Indeed, the myofibres progressively degenerate in ache-/- embryos due to the continuous activity of the *acheR* at the neuromusclular junctions, while we observed a detachment of fibres from the Z lines in DU-exposed larvae, and vacuolization of the muscle at more macroscopic levels. We didn’t observe obvious swimming phenotype on the DU-exposed larvae. These data suggest that the histological defects observed in the muscles translate mainly in the induction of stress, through the up-regulation of chaperons and gene coding for titin binding proteins like tcap involved in sarcomere assembly, and not in a dystrophic condition. Dedicated experiments will be required to observe subtle or long-term defects in swimming behaviour. Uranium and DU increase both the level of *ache* in the zebrafish brain [59,60] and in skeletal muscles (B. Gagnaire, personal communication). The disorganization of muscle fibres could thus be a consequence of *ache* overstimulation or a more generic effect of DU on the neuromuscular junctions that may perturb neurotransmission.

## Conclusion

This study provides a comprehensive analysis of the DU-induced transcriptomic responses in the brain and gonads (testis and ovaries) of adult fish, and the potential transgenerational effects in two developmental stages of their progeny, the two-cells stage and the four-day old larvae. It extends the precedent findings on DU toxicity by giving molecular mechanisms of action of DU and demonstrates that these effects are also observed in the progeny of the exposed fish. The tissue specific DU-induced effects include the perturbation of centromeres organisation in the adult testis, and an impact on the visual perception mechanisms in the brain. The process of cell adhesion is impacted both in DU-exposed adult fish and in their progeny. Our data show that chronic exposure to low dose of DU increases oxidative stress, and suggest that it may induce DNA double-strand breaks, and genomic instability in the zebrafish. These genotoxic effects lead to the increases of somatic maintenance costs [25] and could foster the emergence of cancers and metabolic diseases in both the exposed individuals and in their progeny. We show that DU alters the genetic networks regulated by the upstream regulator *egr1*, a transcription factor deregulated upon carcinogenesis and involved in the response to oxidative stress and the cell-adhesion process. The central role of *egr1* in these mechanisms highlights its potential usage as a biomarker of DU toxicity. The transgenerational DU-induced effects include the dysfunction of mitochondrial activity, the disruption of myofibres in the skeletal muscles and a disorganisation of the neuromuscular junctions. A potential increase of hatching time might also be possible due to the down-regulation of the hatching enzymes *he1a* and *he1b*. The cellular stress is sensed by the induction of protein chaperons like *hsp70*, *hsp70l and dnajb1b* (*hsp40*) in the skeletal muscles. Several outcomes on the fitness of the chronically DU-exposed adults can be proposed such as a decrease in the sperm quality, an impact on visual perception, a potential decrease in swimming ability, an increased hatching time of the progeny and finally an increased occurrence of cancer in the exposed adult and their progeny.

## Supporting information

S1 Fig Hierarchical clustering. (A) Hierarchical clustering of Euclidean distances of all samples and all genes analysed by mRNAseq. Red: low distance (high correlation), yellow high distance (low correlation). *: outliers removed in the subsequent analysis due to high variability. (B) Hierarchical clustering of the Euclidean distances of 22 selected samples after removal of outliers. This analysis was performed on the set of 6140 genes expressed at high level in at least one condition (rlog > 11). C: non-exposed control, U: DU exposed, testis: adult testis, ovary: adult ovary, two-cells: embryos at two-cells stage, brainM: adult brain from males, brainF: adult brain from females, 96 hpf: larvae at 96 hours post fertilization. The replicate numbers are indicated for each sample. (C) Hierarchical clustering of normalized genes expression (rlog > = 11). High expression are displayed in red, moderate expression in white and low expression in blue. C: non-exposed control, U: DU-exposed, testis: adult testis, brain: adult brain (females), two-cells: embryos at two-cells stage, 96 hpf: larvae at 96 hours post fertilization. The replicate number is indicated for each sample.
(TIF)

S2 Fig Venn-diagram of down-regulated genes after DU-exposure in the brain and the testis. (TIF)

S3 Fig Enrichment of disorders in the progeny of the DU-exposed fish. (A) Analysis of the disorders (MESH terms) enriched in the progeny of DU-exposed fish at two-cells stage and 96 hpf. (B) Venn-diagram of human orthologues that characterise human disorders (MESH terms) and that are also differentially expressed in the two-cells stage embryos and the 96 hpf larvae.
(TIF)

S4 Fig Gene Ontology enrichment. (A) Heatmap of adjusted *p*-values in the 96 hpf larvae for the genes involved in response to oxidative stress (GO:0006979, *n* = 63 genes) and in lipid metabolic process (GO:0006629, *n* = 539 genes). The colour code displays the log10(adjusted *p*-value). (B) MA-plot showing the differential expression of genes involved in response to oxidative stress (GO:0006979, *n* = 63 genes), lipid metabolic process (GO:0006629, *n* = 539 genes) and neuron projection regeneration (GO:0050789, *n* = 30) (fold change as DU/C). (C) Quantitative RT-PCR for *egr1* in the 96 hpf larvae.
(TIF)

S1 Table Quality control of paired end reads generated by mRNAseq. The number of reads and the mean quality scores (phred-score, Q) are indicated for each sample. PF: clusters passing Illumina chastity filter. DU: fish exposed to DU.
(XLSX)

S2 Table Enrichment of Gene Ontology terms in adult brain, testis and ovaries from DU-exposed fish. The set of genes used for the analysis and the GO terms are indicated. The *p*-values correspond to the results of the Fisher’s exact-test.
(XLSX)

S3 Table List of genes co-regulated in both adult brain and testis after 10 days of chronic exposure to DU. The log2(fold change) and adjusted *p*-values are indicated for both conditions compared to controls.
(XLSX)

S4 Table Enrichment of Gene Ontology terms at the two-cells stage and in 96 hpf larvae. The set of genes used for the analysis are indicated (cut-off used for the genes selection: log2 fold change > ±1 and adjusted *p*-values < 0.01). The *p*-values correspond to the results of the Fisher’s exact-test.
(XLSX)

S5 Table Enrichment of GO terms with human orthologues. The list of GO terms and the deregulated genes (human annotations) are indicated.
(XLSX)

S6 Table List of human disorders enriched after DU-exposure. The human orthologues of the deregulated zebrafish genes were used for the enrichment (cutoff log2 fold change > ±1 and adjusted *p*-values < 0.01). The MESH terms identifiers, disease name, adjusted *p*-values and genes identity (human annotations) are indicated.
(XLSX)

S7 Table List of the human gene orthologues potentially involved in DU-associated diseases. Human orthologues that characterise the MESH terms and that are also deregulated in the zebrafish datasets at 96 hpf and two-cells stage (cutoff log2 fold change > ±1 and *p*-values < 0.01).
(XLSX)

S8 Table Enrichment of up-stream regulators based on the molecular signature observed in the mRNAseq-datasets. The enrichment of upstream-regulators (transcription factors and chromatin remodelers) was performed by selecting mis-regulated genes and fetching the human orthologues. The condition, the enriched transcriptional regulators as well as the *p*-values from the Fisher’s exact test are indicated.
(XLSX)

S9 Table List of the potential up-stream regulators and their differential expression in the mRNAseq datasets. The zebrafish genes identifiers and the adjusted *p*-values (FDR) are provided. ne: not expressed.
(XLSX)

S10 Table ARRIVE guidelines checklist. (PDF)
