# Supplementary figures and images for: Zebrafish exposure to environmentally relevant concentration of depleted uranium impairs progeny development at the molecular and histological levels
Source: PLoS One. 2017 May 22;12(5):e0177932. doi: 10.1371/journal.pone.0177932 (PMC5439696; doi:10.1371/journal.pone.0177932)

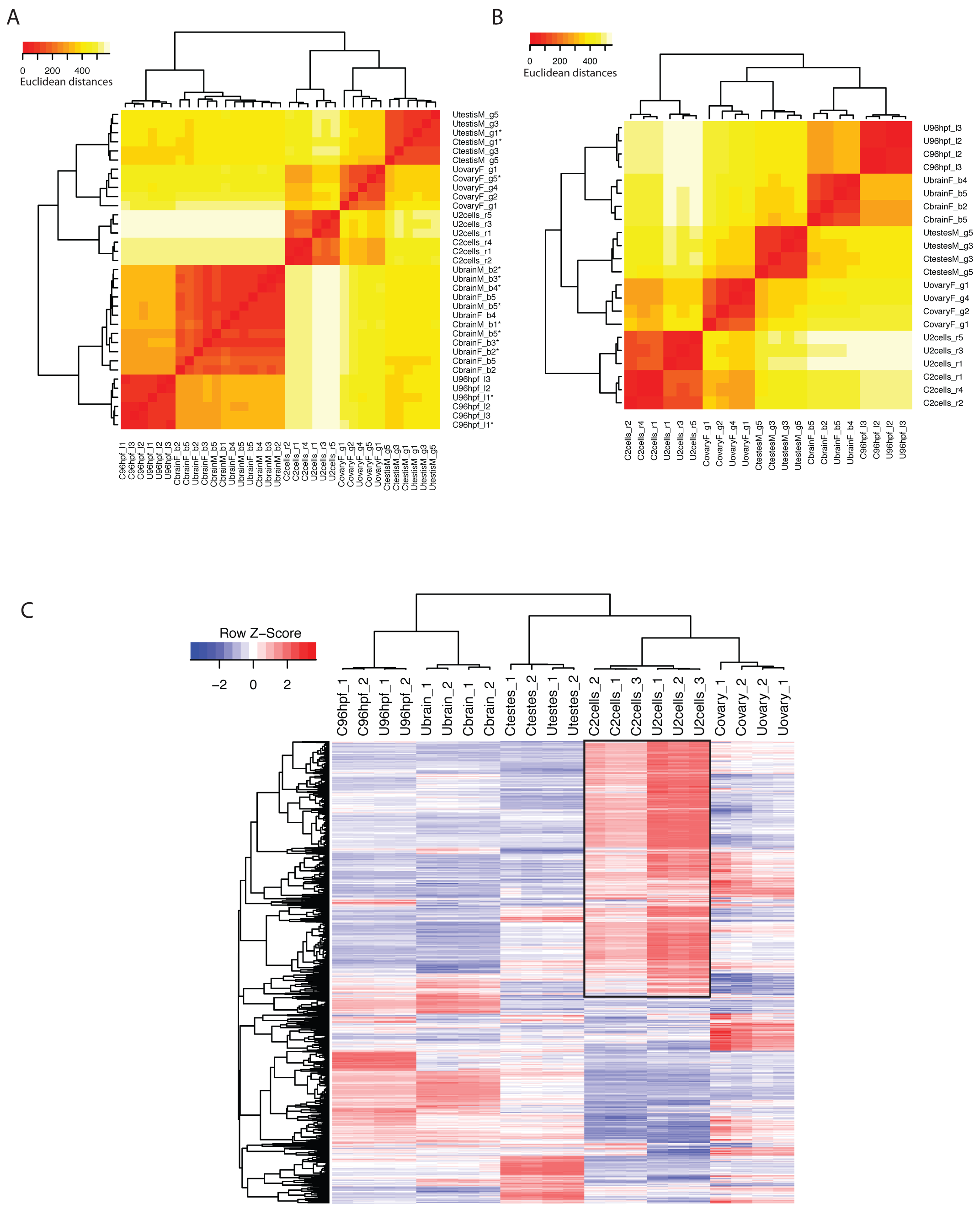

Supplement: S1 Fig — (A) Hierarchical clustering of Euclidean distances of all samples and all genes analysed by mRNAseq. Red: low distance (high correlation), yellow high distance (low correlation). *: outliers removed in the subsequent analysis due to high variability. (B) Hierarchical clustering of the Euclidean distances of 22 selected samples after removal of outliers. This analysis was performed on the set of 6140 genes expressed at high level in at least one condition (rlog > 11). C: non-exposed control, U: DU exposed, testis: adult testis, ovary: adult ovary, two-cells: embryos at two-cells stage, brainM: adult brain from males, brainF: adult brain from females, 96 hpf: larvae at 96 hours post fertilization. The replicate numbers are indicated for each sample. (C) Hierarchical clustering of normalized genes expression (rlog > = 11). High expression are displayed in red, moderate expression in white and low expression in blue. C: non-exposed control, U: DU-exposed, testis: adult testis, brain: adult brain (females), two-cells: embryos at two-cells stage, 96 hpf: larvae at 96 hours post fertilization. The replicate number is indicated for each sample. (TIF) [file pone.0177932.s001.tif]

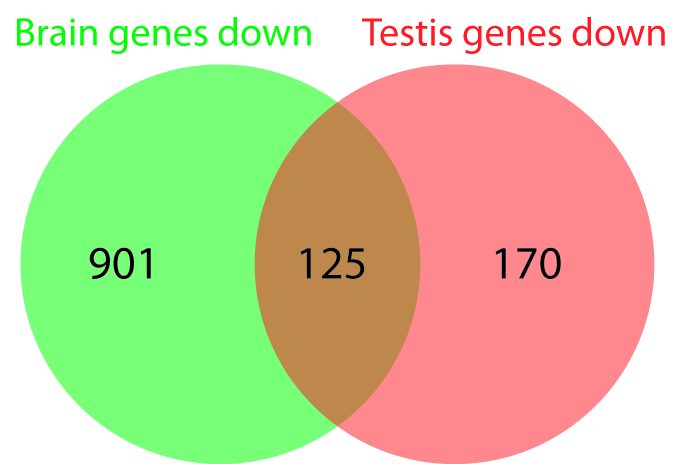

Supplement: S2 Fig — (TIF) [file pone.0177932.s002.tif]

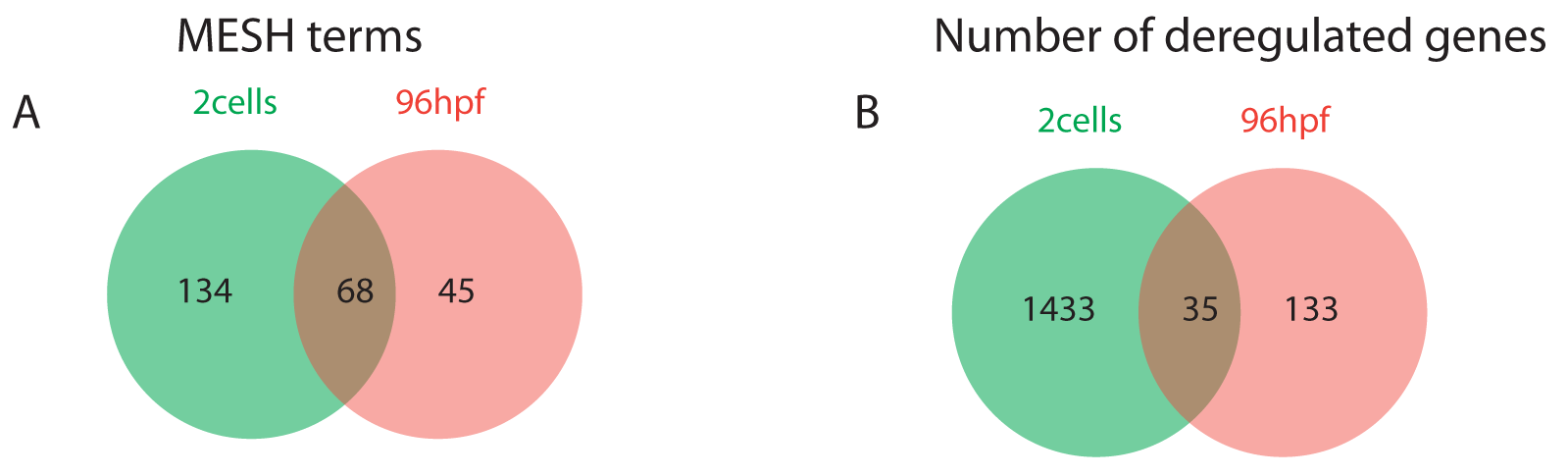

Supplement: S3 Fig — (A) Analysis of the disorders (MESH terms) enriched in the progeny of DU-exposed fish at two-cells stage and 96 hpf. (B) Venn-diagram of human orthologues that characterise human disorders (MESH terms) and that are also differentially expressed in the two-cells stage embryos and the 96 hpf larvae. (TIF) [file pone.0177932.s003.tif]

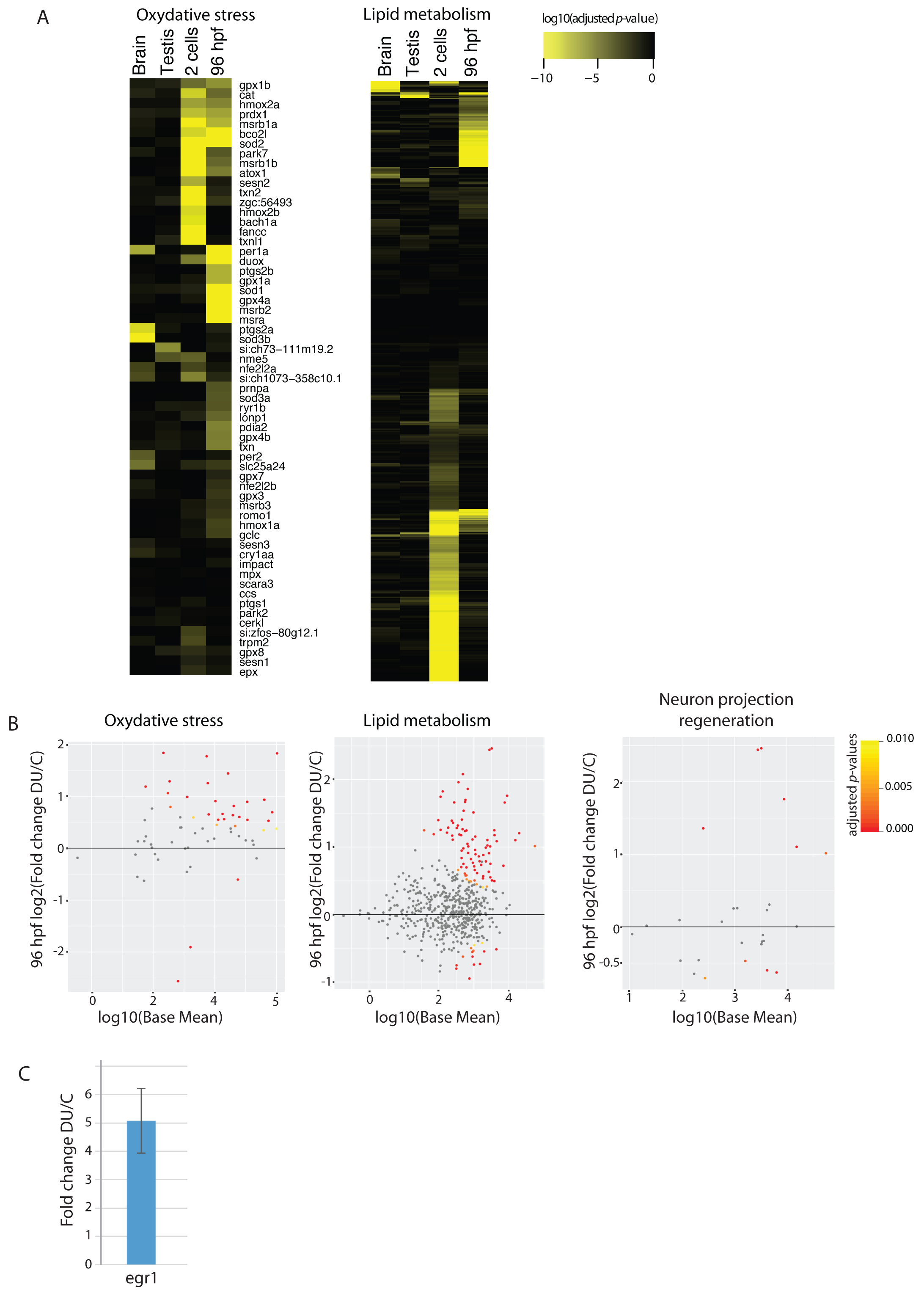

Supplement: S4 Fig — (A) Heatmap of adjusted p-values in the 96 hpf larvae for the genes involved in response to oxidative stress (GO:0006979, n = 63 genes) and in lipid metabolic process (GO:0006629, n = 539 genes). The colour code displays the log10(adjusted p-value). (B) MA-plot showing the differential expression of genes involved in response to oxidative stress (GO:0006979, n = 63 genes), lipid metabolic process (GO:0006629, n = 539 genes) and neuron projection regeneration (GO:0050789, n = 30) (fold change as DU/C). (C) Quantitative RT-PCR for egr1 in the 96 hpf larvae. (TIF) [file pone.0177932.s004.tif]
